# The need for new acutely acting antimigraine drugs: moving safely outside acute medication overuse

**DOI:** 10.1186/s10194-019-1007-y

**Published:** 2019-05-16

**Authors:** Willem Sebastiaan van Hoogstraten, Antoinette MaassenVanDenBrink

**Affiliations:** 1 Dept. of Neuroscience Erasmus University Medical Centre, PO Box 2040, 3000 CA Rotterdam, The Netherlands; 2 Div. of Pharmacology, Dept. of Internal Medicine, Erasmus University Medical Centre, PO Box 2040, 3000 CA Rotterdam, The Netherlands

**Keywords:** Migraine, Medication overuse headache, Chronic migraine, Acute antimigraine drugs, Triptans, Gepants, Ditans, Lasmiditan

## Abstract

**Background:**

The treatment of migraine is impeded by several difficulties, among which insufficient headache relief, side effects, and risk for developing medication overuse headache (MOH). Thus, new acutely acting antimigraine drugs are currently being developed, among which the small molecule CGRP receptor antagonists, gepants, and the 5-HT_1F_ receptor agonist lasmiditan. Whether treatment with these drugs carries the same risk for developing MOH is currently unknown.

**Main body:**

Pathophysiological studies on MOH in animal models have suggested that decreased 5-hydroxytryptamine (5-HT, serotonin) levels, increased calcitonin-gene related peptide (CGRP) expression and changes in 5-HT receptor expression (lower 5-HT_1B/D_ and higher 5-HT_2A_ expression) may be involved in MOH. The decreased 5-HT may increase cortical spreading depression frequency and induce central sensitization in the cerebral cortex and caudal nucleus of the trigeminal tract. Additionally, low concentrations of 5-HT, a feature often observed in MOH patients, could increase CGRP expression. This provides a possible link between the pathways of 5-HT and CGRP, targets of lasmiditan and gepants, respectively. Since lasmiditan is a 5-HT_1F_ receptor agonist and gepants are CGRP receptor antagonists, they could have different risks for developing MOH because of the different (over) compensation mechanisms following prolonged agonist versus antagonist treatment.

**Conclusion:**

The acute treatment of migraine will certainly improve with the advent of two novel classes of drugs, i.e., the 5-HT_1F_ receptor agonists (lasmiditan) and the small molecule CGRP receptor antagonists (gepants). Data on the effects of 5-HT_1F_ receptor agonism in relation to MOH, as well as the effects of chronic CGRP receptor blockade, are awaited with interest.

## Background

The neurovascular disorder migraine is one of the most common diseases worldwide [1, 2]. While the group of headache disorders is one of the top three causes of years lost to disease (YLDs), migraine is responsible for approximately 87% of these YLDs [3]. Migraine treatment can be divided into acutely acting and preventive treatment. The acutely acting treatment can be further subdivided into migraine-specific treatment and analgesics, which are non-specific drugs [4]. Unfortunately, the current acutely acting treatments do not provide adequate relief of migraine symptoms for all patients [4–6] and, when used frequently, can cause the disease to develop into medication overuse headache (MOH) [7–9], a debilitating disorder estimated to be responsible for approximately 2% of all YLDs [10]. MOH is defined as headache for ≥15 days per month in a patient with pre-existing primary headache, while taking acutely acting medication for 3 months and ≥ 10 or ≥ 15 days per month, in case of specific anti-migraine drugs or simple analgesics, respectively [3, 7].

This unmet need for adequate and safe treatment of migraine has resulted in the development of new drugs, among which 5-HT_1F_ receptor agonists such as lasmiditan, and small molecule CGRP receptor antagonists (gepants) [11–13]. Even though uncertainties regarding long-term effects and precise mechanism of action remain [14–17] and the development of some gepants [18–20] was terminated because of pharmacokinetic or safety concerns, the gepants that are still in development and lasmiditan show promising results in terms of efficacy and side-effects [4, 5, 21]. However, their relationship with medication overuse headache has obviously not yet been described because of the novelty of these drugs. For example, the mean duration until onset of MOH for triptans, ergots, and analgesics is 1.7 years, 2.7 years, and 4.8 years, respectively [22]. This makes it impossible to draw conclusions based upon clinical trials regarding the long-term use of gepants and lasmiditan, and MOH, not knowing what the duration until onset, if there is any MOH, might be for these new drugs.

From epidemiological, clinical, and fundamental animal studies, a substantial amount of evidence regarding the pathophysiology of MOH is available [8, 22–26], we will in this review combine this with the current knowledge about the characteristics of CGRP, gepants, and lasmiditan [12, 27–32] in an attempt to generate a relevant hypothesis regarding MOH and these novel acutely acting antimigraine drugs. To achieve this, we will first shortly review the drugs currently used in the treatment of migraine, after which MOH and its pathophysiology will be discussed, to conclude with new acutely acting drugs in development, and how these drugs are expected to relate to MOH.

### Current acutely acting antimigraine drugs

The most commonly used approaches for the acute treatment of migraine have been extensively reviewed from several perspectives [4, 13, 33–35]. These approaches include the administration of ergot alkaloids (ergots), triptans, NSAIDs, and paracetamol. NSAIDs and paracetamol are both effective in the treatment of migraine, but are considered to be non-specific antimigraine drugs, as they are general analgesics [36–38]. The oldest migraine-specific drugs are the ergots, dating back to before 1900 [39, 40]. Even though several ergots have been shown to be effective against migraine, dihydroergotamine (DHE) is the best tolerated of this class. However, DHE still has more adverse effects than the current drugs. Thus, in practice, 5-HT_1B/1D_ agonists (triptans [41]) are most commonly used. However, a significant proportion of migraine patients experiences insufficient relieve of their attacks, and triptans and ergots are contraindicated in patients with increased cardiovascular risk [42–44]. Additionally, frequent use of any acutely acting antimigraine drugs carries a risk for developing MOH. This results in inadequate treatment of the migraine population as a whole.

### Medication overuse headache

As described above, MOH is a disorder with headache for ≥15 days per month in a patient with pre-existing headache, while taking acutely acting medication for ≥3 months according to certain requirements [3]. From a clinical perspective, MOH is present in about 1% of the general population, and develops mainly in patients with pre-existing migraine (ca. 70% of all MOH cases), or tension-type headache [24, 45] with chronic migraine (CM) being a form of migraine with especially high prevalence of MOH [45]. All classes of acutely acting antimigraine drugs are able to cause development of MOH [22, 23], although clinical differences, such as different mean duration until onset of MOH, remain [22]. MOH patients exhibit, in general, several behavioral characteristics that are also seen in substance abuse or drug addiction [46, 47]. This seems to be in accordance with observations regarding the relapse rate after successful treatment. Although this rate is variable across studies from various countries investigating different separate populations (e.g. populations with triptan overuse, opioid overuse, and / or comorbid psychiatric disorders), the majority shows a relapse rate of 25–35% [45, 48]. Research on the pathophysiology of MOH has, until now, developed in mainly two directions. The first being epidemiological and clinical research on MOH patients, the second pertaining to animal models of MOH. Animal models of CM and MOH usually (repeatedly) administer acutely acting antimigraine drugs (e.g. sumatriptan, paracetamol, opioids) to induce MOH [9, 25, 49–51], or apply nitroglycerin (NO donor) [52–54] or an inflammatory soup on the dura mater [55, 56] to induce CM (with features similar to MOH). These models exhibit several phenotypes that relate to CM as well as MOH, such as mechanical hyperalgesia, photophobia, nociceptive behavior, and facial grooming. However, these models are obviously an imperfect representation of the clinical characteristics. For example, a major critique is that these models cause similar phenotypes, but through a completely different mechanism. Although this may be a strong point, it seems to fit with observations in the clinical situation where diverse classes of drugs may cause similar features of MOH. An obvious difference is that MOH only develops in patients with pre-existing headaches, while in the MOH models naïve mice are exposed to the MOH-inducing drugs. Similarities with the clinical disorders and shortcomings of the animal models are extensively reviewed elsewhere [57]. Utilizing an animal model for MOH, it was shown in 2010 that triptans can induce central sensitization in rats, which could possibly function as a basis for MOH [9]. Since then, ample studies have confirmed that chronical application of drugs like paracetamol [51] and opiates [29, 58, 59] have similar effects, which could possibly underlie the pathogenesis of MOH. Two common observations in MOH models are that CGRP expression increases [9, 25, 28, 30] and 5-HT_1B/D_ receptor expression decreases [60, 61] upon prolonged exposure to antimigraine drugs in animal models. Clinical research has shown that 5-HT levels are decreased in patients with MOH [8, 26, 62]. This decrease in 5-HT levels might subsequently upregulate the pronociceptive 5-HT_2A_ expression [63]. Such an upregulation of 5-HT_2A_ expression is also observed in animal models of MOH [51]. Additionally, reduced 5-HT concentrations in animal models resulted in increased amount of CSDs and hyperexcitability in the cortex and the nucleus caudalis of the trigeminal tract [64–66], mimicking clinical observations in patients with migraine and decreased 5-HT levels. Furthermore, these lower 5-HT levels may also increase CGRP expression [45, 63], providing a possible connection between the increased CGRP and decreased 5-HT levels observed in MOH patients. Blocking CGRP receptors with a monoclonal antibody (mAb) has shown to reduce the risk for cutaneous allodynia, which was used as a proxy for MOH in an animal model utilizing nitroglycerin as inducer [27]. This is in accordance with the concept that increased CGRP levels may be involved in the pathogenesis of MOH [67], although it should be kept in mind that other recent studies did not confirm that systemic CGRP levels are increased in medication overuse headache [68, 69]. In conclusion, decreased 5-HT, increased 5-HT_2A_ receptor level and possibly increased CGRP expression seem to be involved in the pathophysiology of MOH, based upon animal research models.

### Prospective acutely acting antimigraine drugs

The development of new acutely acting drugs has mainly been driven by growing understanding of the pathophysiology of migraine, together with the above-mentioned shortcomings of the currently available drugs. For example, small-molecule CGRP receptor antagonists (gepants) [70], specific 5-HT_1F_ receptor agonists [21], TRPV1 receptor antagonists [71–73], EP4 receptor (with PGE2 as ligand) antagonists [74], and glutamate receptor antagonists [13] have all been pursued because of their link to migraine pathophysiology [75]. Some of these were, unfortunately, discontinued because of non-superiority over placebo in clinical trials [4]. Currently, the most promising and clinically advanced candidate drugs are lasmiditan (5-HT_1F_ receptor agonist) [12, 21, 76, 77] and gepants (CGRP receptor antagonists) [31, 70, 78, 79]. Lasmiditan is a specific 5-HT_1F_ receptor agonist, whereas triptans have a higher affinity for the 5-HT_1B/1D_ receptors [12]. This difference in affinity is important because triptans are thought to contract the middle meningeal arteries [80], coronary arteries [43, 81], and increase the blood pressure [82] through their action on the 5-HT_1B_ receptor [42], for which lasmiditan has no affinity at clinically relevant concentrations. Consequently, where sumatriptan has been shown to have the potential to constrict coronary and carotid arteries *in vivo* [44] and in vitro [83], lasmiditan did not possess any vasoconstrictor properties in these studies. Because coronary artery constriction brings a cardiovascular risk and lasmiditan does not constrict the coronary arteries either *in vitro* or in vivo, lasmiditan does not appear to carry the same cardiovascular risk as triptans, which makes it potentially applicable to a wider population. Although it has a lower risk for cardiovascular side effects, lasmiditan may induce central side effects such as dizziness, fatigue, and paresthesia [12, 76]. Simultaneously with the research focusing on the 5-HT_1F_ receptor agonist lasmiditan, multiple gepants (small molecule CGRP receptor antagonists) are currently being developed for the treatment of migraine [70, 84]. The gepants still in development for the acute treatment of migraine, ubrogepant and rimegepant, show a significant effect compared to placebo, although their efficacy relative to other antimigraine treatments remains to be explored [85]. They seem to cause less side effects than existing anti-migraine drugs, but could potentially carry a cardiovascular risk [16] as CGRP is known to possess cardioprotective properties [86]. Additionally, CGRP/calcitonin knock-out animal models have demonstrated to be more susceptible for hypertension when hypertension is triggered [87, 88]. Presently there is not sufficient evidence to determine whether gepants will have side effects on the cardiovascular system. In summary, the two most promising new acutely acting antimigraine drugs are lasmiditan and the gepants, where lasmiditan has a low cardiovascular risk but central side effects and gepants show the least side effects but potentially could carry a cardiovascular risk, although not sufficient evidence to support or refute this concern is available at the moment.

### Pharmacology of lasmiditan, CGRP and MOH

**Fig. 1:**
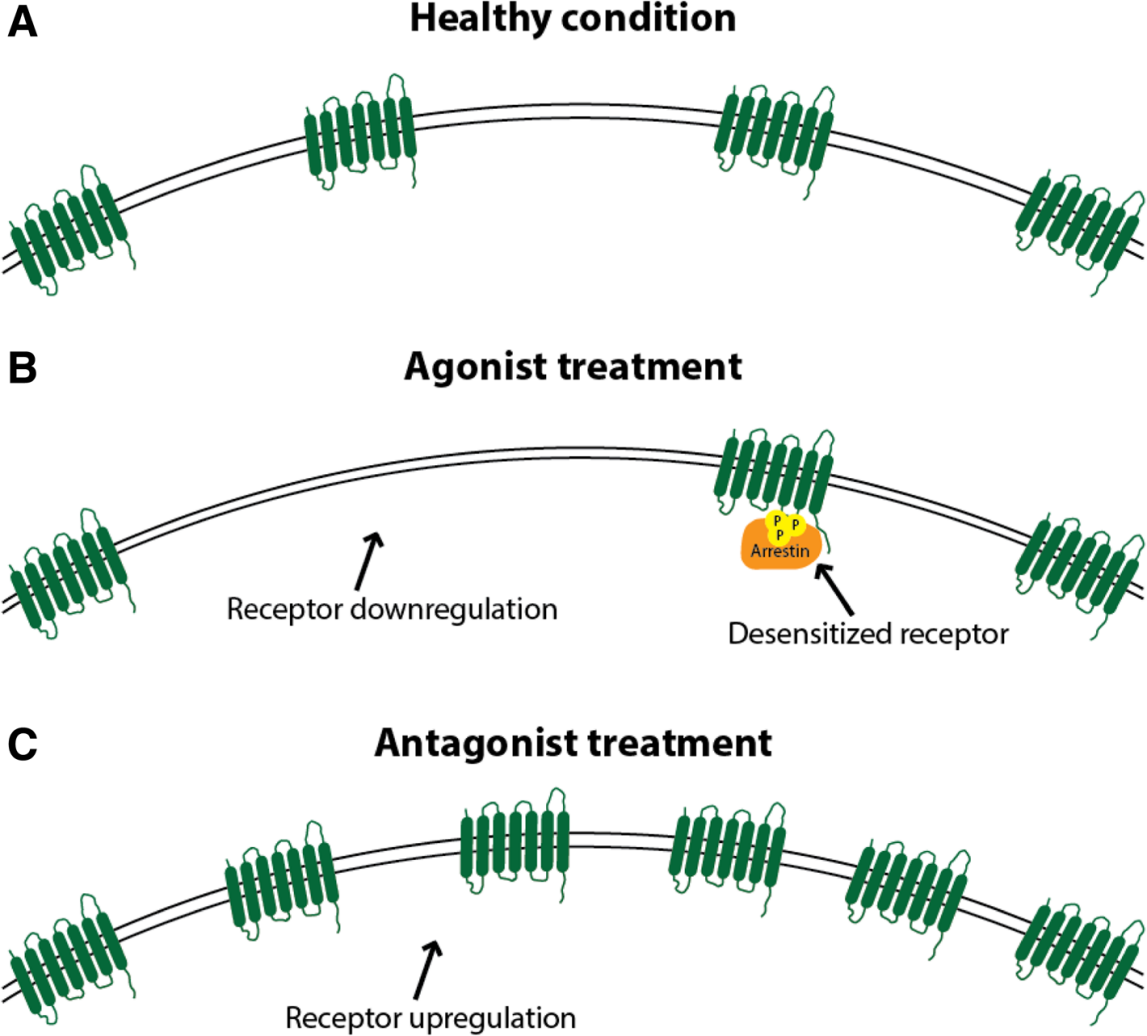
Schematic representation of potential receptor expression changes upon chronic drug use. Receptor expression in the cell membrane in healthy condition (**a**), after prolonged agonist exposure (**b**), and after prolonged antagonist exposure (**c**). After prolonged agonist exposure, downregulation and desensitization (by arrestin binding after phosphorylation by GPCR Kinase) could occur. After prolonged antagonist exposure, receptor upregulation is expected to take place

A question that is of great interest, is whether novel drugs like lasmiditan and the gepants will have the capability to induce MOH. While, as outlined above, the exact mechanisms behind MOH are currently unknown, it makes sense to hypothesize that MOH may have to do with desensitization and / or downregulation of the receptors involved in the drug response. It is likely that treatment with agonists will lead to a receptor desensitization and / or downregulation, while treatment with receptor antagonists will lead to receptor upregulation [89] (Fig. 1), as previously reported in depth for the ß-adrenoceptor agonists used for cardiovascular indications [90]. Besides direct effects on the receptors involved, different classes of drugs leading to MOH may also affect up- or downregulation of the targeted receptor / pathways, potentially leading to a common downstream mechanism inducing MOH. Admittedly, many aspects, such as differential intracellular signaling pathways [91] are still incompletely understood. In addition, migraine patients may have a specific (epi) genetic propensity leading to MOH, which may not be reflected in animal models. While triptans are known to have the propensity of inducing MOH when taken too frequently, it is not known whether selective 5-HT_1F_ receptor agonists, such as lasmiditan, carry the same risk. Theoretically, this could be possible because the 5-HT_1B_, 5-HT_1D_ and 5-HT_1F_ receptors all bind to a G_i/o_–coupled receptor and negatively couple to adenylyl cyclase and, thus, share the same effect: decreased production of cyclic AMP [92, 93]. On the other hand, stimulation of the 5-HT_1F_ (as well as 5-HT_1D_) receptor, which has been described to be present in blood vessels [94], does not constrict these blood vessels, despite the shared second messenger pathway with the 5-HT_1B_ receptor, underlining that not all characteristics of stimulation of certain receptors can be predicted based on their shared intracellular signaling pathways. Clearly, 5-HT_1B/1D_ receptor agonists with a poor potency at the 5-HT_1F_ receptor, such as ergotamine, are also capable of inducing MOH [95], so the 5-HT_1F_ receptor is not required for this phenomenon. There are, to the best of our knowledge, currently no data suggesting that the 5-HT_1F_ receptor would or would not be involved in the generation of MOH, so clinical data on the frequent use of 5-HT_1F_ receptor agonists such as lasmiditan are awaited with interest.

Regarding CGRP receptor blockade, chronic and frequent administration of gepants has been attempted in clinical trials investigating prophylactic treatment of migraine [19, 84, 96, 97], and chronic blockade of the CGRP receptor is also achieved by administration of the monoclonal antibody erenumab. Currently, there are no data suggesting that chronic blockade of the CGRP receptor will induce MOH, although long-term effects of administration of CGRP (receptor) – blocking drugs on CGRP receptor signaling should definitely be studied [98]. While blocking CGRP (receptors) is an effective approach for treating migraine, chronic use could in theory result in an increase of CGRP (receptor) expression. However, it is currently unknown whether expression of CGRP (receptors) will increase or decrease under these circumstances [98]. Furthermore, the hypothesis that CGRP has an indirect and direct positive feedback loop was proposed by Russo in 2015 [15]. This would, in theory, imply that (chronically) blocking CGRP would not be answered with an (over) compensation or upregulation of CGRP receptors. For 5-HT, on the contrary, applying triptans results in a decrease in 5-HT levels. In summary, it will be fascinating to study the consequences of, and potential differences between, the chronic administration of 5-HT receptor agonists and CGRP receptor antagonists.

### CGRP and medication overuse headache

As described above, CGRP is a central component of migraine. Levels of CGRP are increased in animal models of MOH, which is probably reflecting CGRP levels in MOH patients [67–69], and blocking CGRP with an antibody prevents the development of a proxy for MOH in a rodent model [27]. Not only does blocking CGRP (receptors) seem to prevent MOH formation, but also has it been shown to reduce headache in clinical trials of MOH treatment [99–101]. In summary, 1) currently no conclusion can be drawn as to whether CGRP, or CGRP receptor, expression will increase upon blockade of either of the two; 2) blocking the CGRP pathway prevents formation of a proxy of MOH in a rodent model [27]; and 3) reduces headache in clinical trials of MOH treatment [99–101]. Thus, the CGRP pathway seems to be a possible candidate in the safe acute (and preventive) treatment of migraine, maintaining a low risk for MOH development. Possibly, it could even contribute to symptom alleviation in already clinically established MOH. However, the effects of long-term blockade of CGRP or its receptors remain to be investigated properly.

### Other novel acutely acting antimigraine drugs and medication overuse headache

Opposed to current acutely acting antimigraine drugs and drugs acting on the CGRP pathway, the relationship with MOH has not extensively been discussed or investigated for novel acutely acting antimigraine drugs. For example, although lasmiditan has been extensively investigated with regard to risk for cardiovascular side effects and efficacy of migraine treatment as described above, currently no data are available regarding its relation to MOH [102]. To estimate the risk for MOH development in patients using lasmiditan, several aspects of the drug should be considered, as mentioned above in this review. We look forward to novel studies shedding more light on these characteristics of the prospective antimigraine drugs.

## Conclusion

In conclusion, the acute treatment of migraine will certainly improve with the advent of two novel classes of drugs, i.e., the 5-HT_1F_ receptor agonists and the small molecule CGRP receptor antagonists (gepants). Data on the effects of 5-HT_1F_ receptor agonism in relation to MOH, as well as the effects of chronic CGRP receptor blockade, are awaited with interest.

## Abbreviations

5-HT: 5-hydroxytryptamine, serotonin
CGRP: calcitonin gene related peptide
CM: chronic migraine
CSD: cortical spreading depression
DHE: dihydroergotamine:
E4: prostaglandin E2 receptor 4
mAb: monoclonal antibody
MOH: medication overuse headache
NO: nitric oxide
NSAIDs: non-steroidal anti-inflammatory drugs
PGE2: prostaglandin E2
TRPV1: transient receptor potential vannilloid 1
YLDs: years lost to disease
